# Miscellaneous Notices

**Published:** 1856-07

**Authors:** 


### MISCELLANEOUS NOTICES.

*American Dental Convention.*—It will be seen from the following communication, that the second meeting of the above named society will be held on Wednesday, Aug. 6th, in the city of New York. We have reason to believe, from the notices we have seen in several of the Dental Journals, and from what we have heard from various members of the profession, that the meeting will be well attended.

We have on numerous former occasions, dwelt at considerable length upon the benefits which may be derived from free intercommunication between the members of the dental profession, and from associated effort for the advancement of science. Therefore, we do not deem it necessary to say more at the present time than merely to call the attention of our readers to the subject. We trust that the deliberations of the convention will be characterized by a spirit of harmony.—Eds.

*Philadelphia,* May 15, 1856.

Dear Sir—Through the medium of the Dental Journals you have, no doubt, become aware that the "American Dental Convention" assembled, organized, and held its first session in the city of Philadelphia, August 2d, 1855.

In forming this Association, its members were animated by the desire to establish a Society upon a basis broad enough to obtain the united support of the entire profession; and that its platform should be such as to secure to each and every dental practitioner, not only in the United States but the entire world, an opportunity for a liberal expression of views upon the various topics connected with the principles and practice of the profession.

By referring to the report of the proceedings, you will find that throughout the entire session the most perfect unanimity of feeling prevailed, and that the various subjects broached, elicited a free and spontaneous expression of opinion from those present.

The second meeting of the "Convention," will be held in the city of New York, Wednesday, August 6th, 1856. At this, by the desire of the Association, you are respectfully and earnestly invited to be present.

Trusting that you will give the movement your cordial support, I remain,

Yours, respectfully,

J. H. McQuillen, *Cor. Secy.*

*Western Dental Society.*—It will be seen from the following notice sent to the senior editor, that the dentists of the far west, have organized themselves into a society for the advancement of science and the elevation of the dignity of their profession. We wish them abundant success.

*St. Louis,* June, 1856.

*Dear Sir:* An adjourned meeting of the Western Dental Society, is to be held in the city of Chicago, commencing on Wednesday, July 30th, at 10 o'clock, A. M.

The object of the meeting is to continue the work which had so favorable a beginning at the meeting held in this city in April last, by extending the membership and operations of the society over that portion of the west which has not been hitherto embraced in any similar organization.

The invitation is intended to be general, and it is hoped that no one will feel himself neglected in case he does not receive a copy of this circular; but that each and all will interest themselves in the movement, and give it their hearty co-operation and support.

The place of meeting can be ascertained by inquiry at any of the principal hotels in Chicago.

Very respectfully, yours,

C. W. Spalding, *Cor. Sec'y.*

*California Medical Society.*—We have received by mail a copy of the constitution and by-laws of the Medical Society of the State of California.

Among the names of officers and members, we recognize many with whom we were formerly acquainted. Among that number we notice the name of Dr. B. B. Brown, formerly a practitioner of dentistry in St. Louis. We congratulate the officers and members of the Medical Society of the State of California upon the formation of their association.

*India Rubber Base for Artificial Teeth.*—In a previous number of the Journal we took occasion to notice the application of vulcanized india rubber as a base for artificial teeth. A few weeks since we received the following communication from Dr. Mallet, dentist, of Hartford, Conn., giving the result of a series of experiments in the use of this article, which cannot fail to be interesting to our readers; therefore, rather than permit the communication to lay over until our next issue, we introduce it into the editorial department of the present number—the portion assigned to original communications having been closed previously to the receipt of the article.—Eds.

*India Rubber Teeth.*—By Dr. Samuel Mallet, dentist.—Some fugitive notices have from time to time appeared in the public prints regarding the use of hard rubber for dental purposes, but I believe no one has described anything of a practical nature, respecting this article for the purpose named. The idea has been in many minds, that it would be accomplished, but *how* the thing could be done, has been the question.

The first piece of work of the kind, which in any manner resembled a set of teeth, was an upper set; plate, teeth and gums, all of hard rubber, and in one piece, being made by Mr. F. A. Bevins, in the employ of Mr. Goodyear, by moulding an artificial set of teeth in plaster of paris, and filling the whole with sheet rubber, prepared for such purposes. This was then subjected to the influence of steam, and in the process of vulcanizing, a kind of fusion of the rubber took place, which caused every portion of the mould to be filled, making an exact copy of the case of teeth which was used. But notwithstanding the adaptation to the mouth was most perfect, it would not answer for practical purposes, the color would condemn it, and besides, the teeth were not sufficiently dense to endure the friction caused by mastication. But subsequent experiments warrant a hope this difficulty will yet be overcome.

About this time, Mr. Goodyear, now in France, sent to this country a partial set of teeth, the plate, gums and clasps being composed of rubber, in connection with plain mineral teeth, inserted in the rubber, somewhat like gutta percha work.

About this time, with the aid of Mr. Bevins, an experienced workman in rubber, I made a case of teeth in the same manner. But we were satisfied that this mode of doing the work would not go into very general use, unless the color of the gum could be made more closely to resemble nature, it being about that of mahogany. Many experiments were made to improve the color of the rubber, but they were unsuccessful in bringing it to a life resemblance. We then hit upon a plan of using single gum teeth, and soon a denture was produced which has been worn since that time with much satisfaction.

The durability of the work has surprised me; although the ordinary teeth were used with no attachments but such as were made by the pins and the angles of the teeth, yet I do not see, that they are more liable to loosen from the case than they are when made in the ordinary way of inserting them on gold plate, this is more than I at first expected of the work. The rubber has peculiar qualities. It has wonderful tenacity, when it is under the action of steam, it expands and seeks vent, forcing itself between the particles lying on the surface of the object with which it comes in contact. To show the strength of it I will name, that I took the incisor teeth of a case between my thumb and fingers and attempted to break them from their beds, (these were gum teeth,) but could not without breaking the plate in another place first. I am now using block teeth for this purpose, and I am satisfied that with the plan of making them which I have designed for this work, they will possess a degree of beauty and strength, which cannot be excelled. The work is performed in the following manner: A plate is fitted to the mouth in the ordinary way, and if it is desirable to avoid swaging, gutta percha may be used for the purpose of fitting. The antagonizing model having been taken and the teeth fitted as for other work; with the plate and teeth, by the means of wax, a perfect model is made, in outline of what it is desired the case should be when completed. It is important that this should be exact. A wax rim is made, enclosing the edges of the teeth or blocks on the labial and lingual sides to represent the band and backings of the teeth.

The model is now to be moulded, the mould being made in sections, so as to fit together very accurately; the teeth are then removed from the model and placed in their respective sockets in the moulds, the rubber being placed in the mould in sufficient quantity to fill it when vulcanised.

The mould being securely enveloped, it is submitted to the process of vulcanising by confining it in a steam chamber for several hours; this done the work is ready to be removed from the mould and polished.

Teeth should be made expressly for this work. There is no difficulty in making single teeth or blocks, which shall be as strong as desirable. I have the block made with two or more excavations about one-eighth of an inch deep on the lingual side, with projections, and in these excavations are pins sufficiently long to allow them to be bent into any desirable shape. The block is also made thicker on this side than for ordinary work, tapered to nearly an edge which is beveled, so that an arch is formed conforming in this particular to nature. In the ends of the blocks where they join together, large corresponding holes are made, into which the rubber flows, making connecting pins of great strength; the rubber also flows around the edges of the blocks, forming the rim on the labial and lingual sides and filling the notches even with the block, so that when the case is finished, there is a uniform surface of rubber and porcelain, differing in appearance only in color, in other respects the blocks do not differ from those which are ordinarily made.

There is a marked difference between the adaptation of metal and hard rubber for plates, and that very much in favor of the latter article. There seems to be more congeniality between the animal system and rubber than is found between it and the various metals used in dentistry. The slight elasticity which it possesses is very agreeable, so different from the rigidity of metallic plates, that it cannot fail to be popular.

---

*Adjustable Punch.*—I have an instrument for punching the holes in the backings of teeth, which is adjusted by the pins of the tooth, for which we wish to punch. One punch is movable, and with the tooth it can be set in an instant, so that the holes punched will exactly correspond to the pins in the tooth, this is an instrument of which every dentist has felt the need. I showed it last November to Dr. Clark, of New Orleans, Drs. C. C. Allen, and John Allen and A. Jones, Esq., of New York, who all approved of it much, and some of them remarked that "it was one of the most complete things, for a small one, which they had seen for a great while." There have been unforeseen reasons why it has not been made known and prepared for the use of the profession.

Mr. Chevalier, of New York, has it now in hand, and will soon be ready to fill orders. A full description of the instrument, accompanied with an engraving, will be furnished for the Dental Journal. S. Mallet.

*Osseous Union of Teeth.*—We have frequently had occasion to notice examples of osseous union of temporary teeth, very many having fallen under our own immedieate observation, but with the permanent teeth occurrences of this kind are altogether less frequent. Indeed, until recently, we never saw but two cases of the kind. These are noticed in "Principles and Practice of Dental Surgery." But within the last few weeks, we have received from Dr. S. Mallet, dentist, of New Haven, Ct., an upper central and lateral incisor belonging to second dentition, perfectly united from their cutting edges to within nearly an eighth of an inch of the extremity of their roots.

Soon after the receipt of the above described specimen, we received the following note from Dr. Goodwin, of North Carolina, enclosing another very interesting example of osseous union of a lower permanent lateral and supernumerary tooth.—Sen. Ed.

*Elizabeth City, N. C.*, Nov. 8th, 1855.

Dr. C. A. Harris: *Dear Sir*—The enclosed specimen of osseous union between the fangs of a right lateral incisor, and a ***supernumerary lateral incisor***, which I recently extracted from the mouth of a patient, seems to me to possess some claims to be considered a novel "freak of nature," as it seems to combine two departures from established law in the same case. Viewing the specimen as rare as curious, I am induced to forward it to you; believing that your interest in whatever concerns the profession, will impart to this little curiosity a value, that will entitle it to be classed among the many singular phenomena, that are constantly presenting themselves to our notice. The *double* deviation which is here found, has suggested the idea of presenting the specimen to you, being the first that I have ever encountered in my practice, though with others it may be of frequent occurrence. Hoping that you may find something of interest in the specimen, I remain yours,

Very respectfully, J. B. Godwin.

---

*Supenumerary Teeth.*—The senior editor begs to acknowledge the receipt of four supernumerary teeth, two from Dr. Bessent, of Concord, N. C., and two from Dr. Thompson, of Providence, R. I. The first two were extracted from behind and between the upper central incisors; the other two were taken from between the first and second upper molars—one from each side.

---

*Introductory Lecture.*—We are indebted to J. C. Clendon, M. R. C. S., dentist, for a very interesting and able introductory lecture, which he delivered at the commencement of his last course at the Westminster Hospital, London, on the diseases of the teeth. We would be glad to make some extracts from it, but are prevented by want of room.

---

*Omitted.*—Although the present No. of the Journal does not fall short of the usual limits, it was nearly filled before the editors were aware of the fact, consequently they are compelled to omit several editorial articles designed for the present issue.

